# Metformin rescues rapamycin-induced mitochondrial dysfunction and attenuates rheumatoid arthritis with metabolic syndrome

**DOI:** 10.1186/s13075-020-02174-3

**Published:** 2020-04-10

**Authors:** Eun Kyung Kim, Hong Ki Min, Seon-Yeong Lee, Da-Som Kim, Jun-Geol Ryu, Hyun Sik Na, Kyoung Ah Jung, Jeong Won Choi, Sung-Hwan Park, Mi-La Cho

**Affiliations:** 1 Rheumatism Research Center, Catholic Institutes of Medical Science, College of Medicine, The Catholic University of Korea, 222, Banpo-Daero, Seocho-gu, Seoul, 06591 Republic of Korea; 2 Laboratory of Immune Network, Conversant Research Consortium in Immunologic Disease, College of Medicine, The Catholic University of Korea, Seoul, Republic of Korea; 3 Division of Rheumatology, Department of Internal Medicine, Konkuk University Medical Center, Konkuk University School of Medicine, Seoul, Republic of Korea; 4 Division of Rheumatology, Department of Internal Medicine, Seoul St. Mary’s Hospital, College of Medicine, The Catholic University of Korea, Seoul, 137-070 South Korea

**Keywords:** Rheumatoid arthritis, Obesity, Mitochondria, Metformin, Rapamycin

## Abstract

**Background:**

Rapamycin, an inhibitor of the serine/threonine protein kinase mTOR, is an immunosuppressant used to treat renal transplant recipients, but it can cause endothelial and mitochondrial dysfunction. Metformin is used for the treatment of type 2 diabetes and was reported to exert therapeutic effects against rheumatoid arthritis and obesity by improving mitochondrial dysfunction via the activation of fibroblast growth factor 21. We investigated the therapeutic effects of rapamycin–metformin combination therapy in obese mice with collagen-induced arthritis (CIA).

**Methods:**

Mouse embryonic fibroblasts were treated with rapamycin, metformin, or rapamycin–metformin, and their respiratory level and mitochondrial gene expression were assayed. Mice were fed a high-fat diet, immunized with type II collagen, and subsequently treated with rapamycin–metformin daily for 10 weeks.

**Results:**

Rapamycin-treated cells exhibited dysfunction of mitochondrial respiration and decreased mitochondrial gene expression compared with rapamycin–metformin-treated cells. Moreover, rapamycin–metformin reduced the clinical arthritis score and the extent of histological inflammation and improved the metabolic profile in obese mice with CIA. Rapamycin–metformin enhanced the balance between T helper 17 and regulatory T cells in vitro and in vivo.

**Conclusions:**

These results suggest that rapamycin–metformin is a potential therapeutic option for autoimmune arthritis.

## Background

Rheumatoid arthritis (RA) is an autoimmune form of arthritis that induces chronic joint inflammation and cartilage damage. RA can also lead to synovial hyperplasia and induces the production of proinflammatory cytokines [1]. Currently, RA is treated with non-steroidal anti-inflammatory drugs, glucocorticoid, and disease-modifying antirheumatic drugs [2]. However, these drugs have side effects, such as bone marrow suppression, reactivation of tuberculosis, hypertension, and renal dysfunction, which hamper the long-term maintenance of therapeutic efficacy [3]. Rapamycin, an inhibitor of the serine/threonine protein kinase mTOR, is used to prevent acute rejection in renal transplant recipients [4]. mTOR acts as a pathological signal for several renal diseases, including glomerular disease, polycystic kidney disease, and renal cancer, and mTOR inhibitors were shown to have therapeutic effects in some renal diseases [5, 6]. However, rapamycin can cause metabolic disorders, such as hyperlipidemia and vascular and mitochondrial dysfunction [7, 8]. Mitochondria produce adenosine triphosphate (ATP) and regulate cellular oxidative stress. Mitochondrial dysfunction exacerbates inflammation and oxidative stress and is associated with the proliferation of fibroblast-like synoviocytes (FLSs) in patients with RA [9, 10]. Although an mTOR inhibitor induced mitochondrial dysfunction, it showed therapeutic effects via suppression of osteoclastogenesis in a mouse model of RA [11]. Furthermore, the addition of an mTOR inhibitor to methotrexate yielded results superior to those obtained with methotrexate monotherapy with regard to the achievement of the American College of Rheumatology 20 response in patients with RA [12].

Metformin is an oral biguanide antidiabetic drug that is effective in patients with type 2 diabetes. It inhibits the inflammatory response by decreasing the T helper 17 (Th17) cell population via downregulation of STAT3 activation and attenuates colitis and collagen-induced arthritis (CIA) in mice with high-fat diet (HFD)-induced obesity [13, 14]. Although metformin reduces ATP production by inhibiting mitochondrial complex I, it activates AMPK, a downstream signaling pathway of the mitochondrial respiratory chain complex, and is involved in intracellular oxygen redistribution [15]. Metformin also showed a regulatory effect on mitochondrial function in a mouse model of inflammation-associated tumors [16].

We hypothesized that combined treatment with rapamycin and metformin would attenuate CIA while maintaining mitochondrial function. Therefore, we evaluated the therapeutic effects of rapamycin–metformin in obese mice with CIA by assaying the mitochondrial function, the metabolic profile, and the CD4^+^ T cell population.

## Methods

### Animals

DBA1/J, C57BL/6 (B6, H-2K^b^), and BALB/c (B/c, H-2k^d^) mice at 8–10 weeks of age (Orient Bio, Gwangju, South Korea) were maintained in groups of five in polycarbonate cages in a specific pathogen–free environment. They were fed chow providing 60 kcal derived from fat or standard mouse chow (Ralston Purina, St. Louis, MO, USA) and water ad libitum. All experimental procedures were approved by the Animal Research Ethics Committee of the Catholic University of Korea (approval number, CUMC 2017-0163-03). All animals were treated and euthanized in accordance with the Catholic University of Korea Guidelines on the Use and Care of Animals.

### Alloreactive T cell responses in vitro

In the mixed lymphocyte reaction assay, cells of 2 × 10^5^ CD4^+^ T cells isolated from the spleens of C57BL/6 mice (responders) were cultured with 2 × 10^5^ irradiated (2500 cGy) BALB/c splenic APC (syngenic stimulator) or C57BL/6 (B6) splenic APC (allogeneic stimulator) for 4 days. Responder cells were cultured in the absence or presence of rapamycin 1 nM and 100 nM. The cells were pulsed with 1 μCi tritiated thymidine (NEN Life Science Products Inc., Boston, MA) 18 h before harvesting and counted using an automated harvester (PHD Cell Harvester; Cambridge Technology, Inc., Cambridge, MA, USA).

### Enzyme-linked immunosorbent assays

The interleukin (IL)-17, IL-6, and tumor necrosis factor-α (TNF-α) concentrations were measured by sandwich enzyme-linked immunosorbent assays (ELISAs; R&D Systems, Minneapolis, MN, USA) in cell culture supernatants. The absorbance at 405 nm was measured using an ELISA microplate reader (Molecular Devices, San Jose, CA, USA). Cytotoxicity was evaluated by 3-(4,5-dimethylthiazol-2-yl)-2,5-diphenyltetrazolium bromide (MTT) assays.

### Measurement of immunoglobulin G concentrations

The immunoglobulin G (IgG) concentrations were measured using mouse IgG ELISA quantitation kits (Bethyl Laboratories, Montgomery, TX, USA) in splenocyte culture supernatant.

### Flow cytometry

Before intracellular staining, cells were stimulated with 25 ng/ml phosphomolybdic acid (Sigma-Aldrich), 250 ng/ml ionomycin (Sigma-Aldrich), and Golgi Stop (BD Biosciences, San Diego, CA, USA) in 5% CO_2_ at 37 °C for 4 h. Cells were stained with peridinin chlorophyll protein complex–conjugated anti-CD4 and allophycocyanin (APC)-conjugated anti-CD25 antibodies (BD Pharmingen, BD Biosciences), and then with a phycoerythrin (PE)-conjugated anti-Foxp3 antibody (eBioscience, San Diego, CA, USA), followed by fixation and permeabilization using a Cytofix/Cytoperm Plus Kit (BD Biosciences) according to the manufacturer’s instructions. The samples were analyzed using a FACSCalibur instrument (BD Pharmingen, BD Biosciences).

### Analysis of mitochondrial membrane potential

NIH3T3 cells were cultured in eight-well chamber slides in the presence or absence of rapamycin–metformin. JC-1 dye was added, and the cells were incubated for 15 min at 37 °C. JC-1–labeled cells were washed in phosphate-buffered saline (PBS), and images were acquired using a confocal laser scanning microscope (LSM 510 Meta; Zeiss, Gottingen, Germany). Mouse splenocytes were placed in 24-well plates and treated with rapamycin. The cells were harvested, JC-1 dye was added, and the cells were incubated for 15 min at 37 °C and analyzed using a CytoFLEX flow cytometer (Beckman Coulter, Brea, CA, USA).

### Confocal microscopy

NIH3T3 cells were stained with MitoTracker Red CMXROS (Molecular Probes, Eugene, OR, USA) for 30 min at 37 °C, washed with PBS, fixed with methanol and acetone, washed, and blocked with normal goat serum. The cells were subsequently stained at 4 °C overnight with a monoclonal anti-α-tubulin antibody (Sigma-Aldrich) and 4′,6-diamidino-2-phenylindole (DAPI) to stain nuclei. The mean fluorescence intensity (MFI) was measured using LSM 510 Meta software (Carl Zeiss, Oberkochen, Germany). For analyses of T helper cell populations, spleen tissue sections (7 μm thick) were fixed and stained with Alexa 488–conjugated anti-CD4, PE-conjugated anti-IL-17, APC-conjugated anti-CD25, and PE-conjugated anti-Foxp3 antibodies (eBioscience). The stained sections were visualized by confocal microscopy (LSM 510 Meta; Carl Zeiss).

### Oxygen consumption rate

An XF24 Extracellular Flux Analyzer (Seahorse Bioscience, Chicopee, MA, USA) was used to measure the cellular oxygen consumption rate. NIH3T3 cells were plated at 2 × 10^4^ per well in XF 24-well culture microplates, washed, and cultured in XF assay medium in a non-CO_2_ incubator. Mitochondrial electron transport was assayed by sequential injections of 2 μM oligomycin, 0.3 or 3 μM carbonyl cyanide 4-(trifluoromethoxy) phenylhydrazone, and 5 μM rotenone/antimycin A.

### Quantitative real-time polymerase chain reaction

RNA was extracted using TRIzol reagent (Molecular Research Center, Inc., Cincinnati, OH, USA). cDNA was synthesized using the Superscript Reverse Transcription System (TaKaRa, Shiga, Japan), and quantitative real-time polymerase chain reaction (PCR) was performed using LightCycler FastStart DNA Master SYBR Green I (TaKaRa) according to the manufacturer’s instructions. The primer sequences for PCR were designed using Primer Express (Applied Biosystems, Foster City, CA, USA) and were as follows: *ndufb5* (forward: TCC CAG AAG GCT ACA TCC CT, reverse: ATT CCG GGC GAT CCA TCT TG), *uqcrb* (forward: TCA AGC AAG TGG CTG GAT GG, reverse: TCA GGT CCA GGG CTC TCT TA), *cox5b* (forward: ATG GGT CCA GTC CCT TCT GT, reverse: GCT TCA AGG TTA CTT CGC GG), *cycs* (forward: AAT CTC CAC GGT CTG TTC GG, reverse: GGT CTG CCC TTT CTC CCT TC), and *β-actin* (forward: GAA ATC GTG CGT GAC ATC AAA G, reverse: TGT AGT TTC ATG GAT GCC ACA G). The mRNA levels were normalized relative to that of β-actin.

### Induction of arthritis and HFD

Chicken type II collagen (CII) immunization was performed in DBA/1J mice. Mice were immunized intradermally via the tail with 100 μg CΙΙ (Chondrex Inc., Redmond, WA, USA) dissolved overnight in 0.1 N acetic acid (4 mg/ml) in complete or incomplete Freund’s adjuvant (Chondrex Inc.). A booster injection was administered 14 days after the primary immunization. The arthritis severity score in the joints was determined twice weekly, and the arthritis score was recorded as the sum of the scores on a scale of 0–4. The mice in the HFD group were fed mouse chow containing 60 kcal derived from fat at the time of primary immunization. The arthritis score index for the disease severity was as follows: 0, no evidence of erythema or swelling; 1, erythema and mild swelling confined to the midfoot (tarsal) or ankle joint; 2, erythema and mild swelling extending from the ankle to the midfoot; 3, erythema and moderate swelling extending from the ankle to the metatarsal joints; and 4, erythema and severe swelling encompassing the ankle, foot, and digits. The maximum possible score per mouse was 16.

### Metformin and rapamycin treatment

Metformin and rapamycin were obtained from Sigma-Aldrich and dissolved in saline. Mice were orally administered 50 mg/kg metformin and/or 1 mg/kg rapamycin daily for 10 weeks starting 7 days after the first immunization. Control mice received saline.

### Histological analysis

Histological analysis was performed to determine the extent of joint damage. Mouse joint tissues were fixed in 4% paraformaldehyde, decalcified in Calci-Clear Rapid (National Diagnostics, Atlanta, GA, USA), embedded in paraffin, and sectioned. The sections were deparaffinized using xylene and dehydrated through an alcohol gradient. Endogenous peroxidase activity was quenched with methanol–3% H_2_O_2_, and the sections were stained with hematoxylin and eosin or safranin O.

### Immunohistochemistry

Immunohistochemistry was performed using a Vectastain ABC Kit (Vector Laboratories, Burlingame, CA, USA). Tissue sections were incubated overnight at 4 °C with primary antibodies against IL-1β, IL-6, IL-17, and TNF-α, probed with biotinylated secondary antibody, and stained with streptavidin–peroxidase complex for 1 h. DAB chromogen (Dako, Carpinteria, CA, USA) was added as a substrate, and the samples were visualized by microscopy (Olympus, Center Valley, PA, USA). Immunohistochemistry was performed on tissue sections of all mice (*n* = 5) of 3 groups. Three slides were prepared for each sample per mice, and each slide was taken at least 500 μm apart. Immunostained sections were examined by a photomicroscope (Olympus, Tokyo, Japan). The number of positive cells was counted at high-power field (magnifications × 400) with the aid of Adobe Photoshop software and averaged 3 randomly selected fields per tissue section. Each cytokine-positive cells in the total cell number were counted and graphed.

### Serum biochemical analyses

Blood samples were collected from mice at 10 weeks and stored at − 70 °C until use. The serum levels of glucose, triglyceride, free fatty acids, aspartate aminotransferase (AST), and alanine aminotransferase (ALT) were measured using kits from Asan Pharmaceutical Co. (Hwangseong-gi, Gyeonggi-do, Korea). The serum levels of the indicated factors were measured using a Hitachi 7600 analyzer (Roche, Basel, Switzerland).

### Glucose and insulin tolerance tests

For insulin tolerance testing, nonfasted mice were injected intraperitoneally with insulin (1 U/kg body weight). For glucose tolerance testing, mice were fasted overnight and injected intraperitoneally with glucose (1 g/kg body weight).

### Statistical analysis

Results are presented as means ± standard deviations or means ± standard errors of the mean. Data were analyzed by Student’s *t* test or the Mann–Whitney *U* test using Prism 5 software (GraphPad, La Jolla, CA, USA). In all analyses, *P* < 0.05 (two-tailed) was taken to indicate statistical significance.

## Results

### Rapamycin reduced T cell alloreactivity and proinflammatory cytokine levels and induced mitochondrial dysfunction, in vitro

**Fig. 1:**
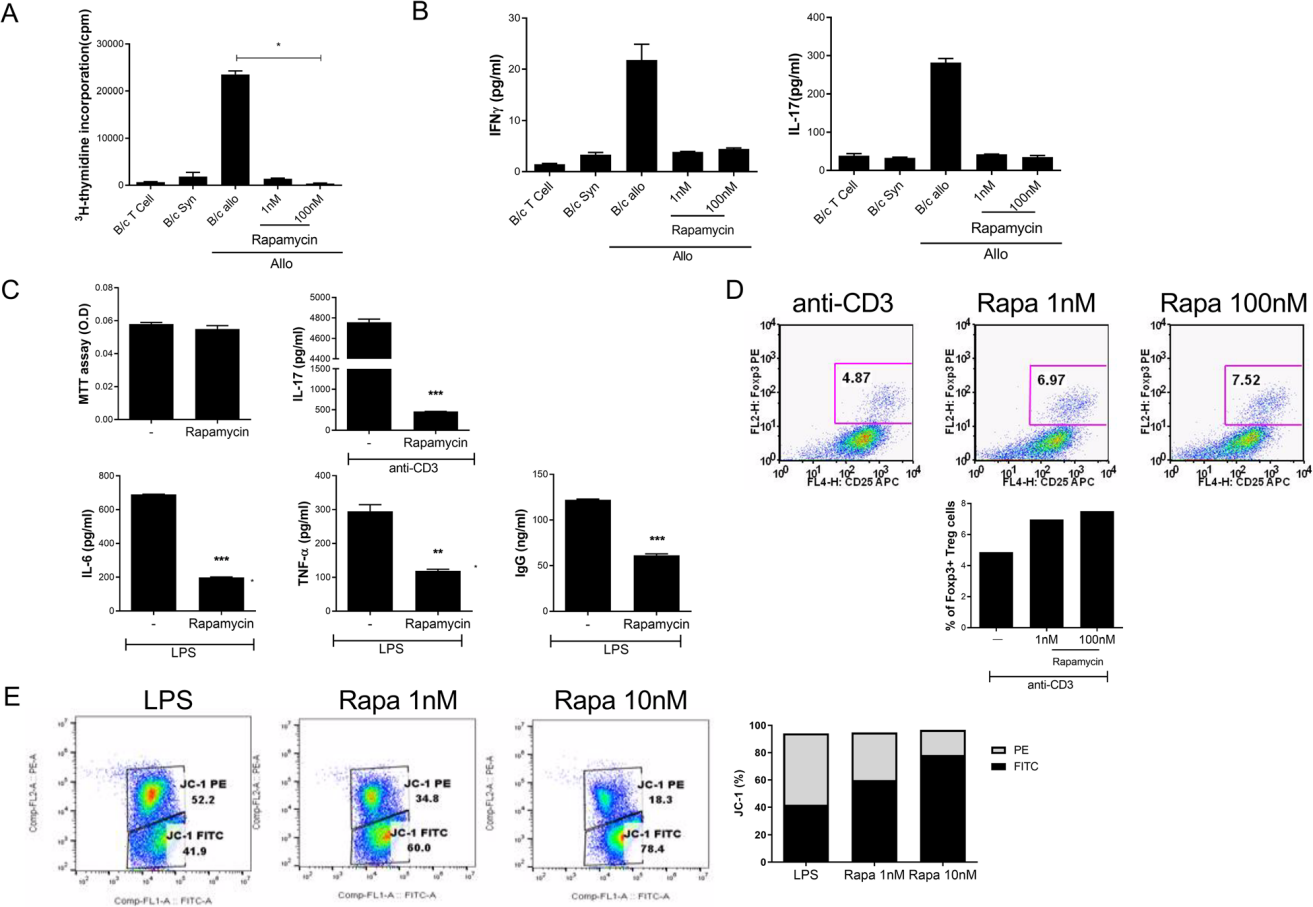
Rapamycin suppresses T cell alloreactivity, proinflammatory cytokines, and production of immunoglobulin and induces mitochondrial dysfunction in vitro. **a** In a mixed lymphocyte reaction assay, B/c splenic T cells (responders) were incubated with irradiated B/c splenic antigen-presenting cells for 4 days. Responder cells were cultured in the presence or absence of rapamycin. **b** IFN-γ and IL-17 levels in the supernatants, as measured by enzyme-linked immunosorbent assay (ELISA). **c** IL-17, IL-6, TNF-α, and IgG concentrations in culture supernatants in the presence of anti-CD3 (0.5 μg/ml) or lipopolysaccharide (100 ng/ml) with rapamycin 100 nM, as determined by ELISA. **d** The Treg population, as determined by flow cytometry. **e** JC-1 populations in isolated splenic cells, as determined by flow cytometry. The data are representative of at least three independent experiments. Data represent the mean ± SD of three independent experiments. **P < 0.01 and ***P < 0.001

To investigate the effects of rapamycin on T cell alloreactivity, we examined its effects on CD4^+^ T cell proliferation in vitro. CD4^+^ T cells from normal B/c mice were cultured with irradiated syngeneic B/c allo splenic antigen-presenting cells treated with or without rapamycin. Alloreactive T cell proliferation was lower in the rapamycin-treated group (rapamycin 100 nM 365.33 ± 97.62 cpm vs. B/C allo 23,510.33 ± 740.66 cpm; *P < 0.01) (Fig. 1a). Rapamycin treatment also reduced the interferon-γ and IL-17 levels in the culture supernatant group (IFN-γ: rapamycin 100 nM 1.44 ± 0.2 pg/ml vs. B/C allo 21.80 ± 3.07 pg/ml, IL-17: rapamycin 100 nM 34.86 ± 4.36 pg/ml vs. B/C allo 281.90 ± 10.70 pg/ml) (Fig. 1b). Next, mouse splenocytes were incubated in the presence of anti-CD3 antibody or lipopolysaccharide with or without rapamycin for 3 days. Rapamycin treatment reduced the levels of IL-17, IL-6, TNF-α, and IgG (IL-17: rapamycin 100 nM 458.17 ± 2.57 pg/ml vs. anti-CD3 4758.73 ± 29.58 pg/ml, IL-6: rapamycin 100 nM 199.23 ± 2.54 pg/ml vs. anti-CD3 691.22 ± 0.421 pg/ml, TNF-α: rapamycin 100 nM 119.2 ± 4.6 pg/ml vs. anti-CD3 295.07 ± 19.55 pg/ml, IgG: rapamycin 100 nM 61.38 ± 1.56 pg/ml vs. anti-CD3 122.22 ± 0.69 pg/ml) (Fig. 1c). In addition, the regulatory T cell (Treg) population was increased following treatment with rapamycin (Fig. 1d). Rapamycin reduced the mitochondrial membrane potential in a dose-dependent manner (Fig. 1e). Although rapamycin exerted an anti-inflammatory effect and modulated T cells, it induced mitochondrial dysfunction.

### Mitochondrial dysfunction was exacerbated by rapamycin

**Fig. 2:**
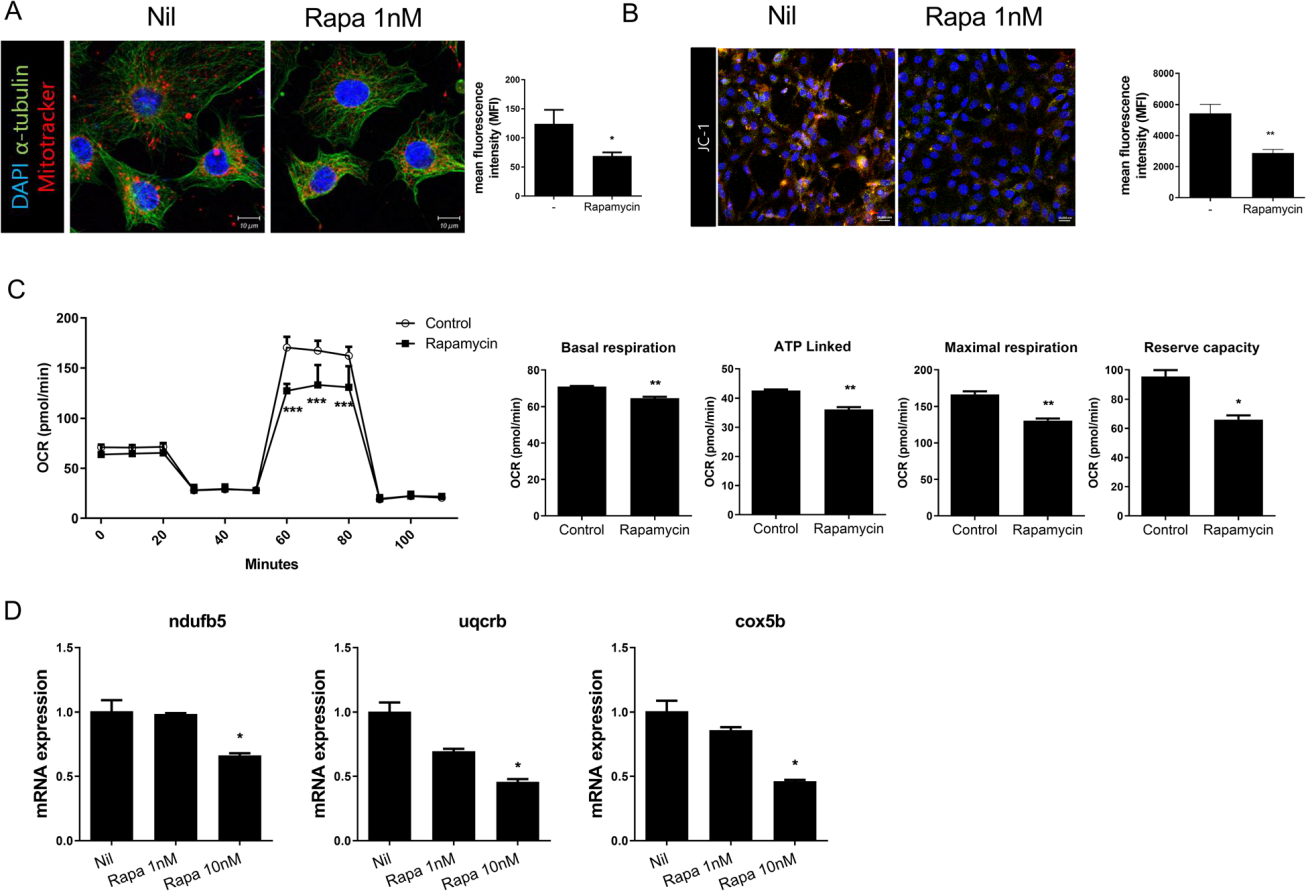
Mitochondrial dysfunction induced by rapamycin. **a** NIH3T3 cells were stained with MitoTracker Red CMXROS (red), anti-α-tubulin (green), and 4′,6-diamidino-2-phenylindole (DAPI; nuclei, blue), and the mitochondrial was quantified on confocal micrographs. **b** Mitochondrial membrane potential was measured using JC-1 dye and confocal microscopy. **c** Oxygen consumption rates (OCR) in the presence and absence of rapamycin. Oligomycin 2 μM, FCCP 3 μM, antimycin A 5 μM, and rotenone 5 μM were treatment in cells for OCR measurement. **d** Mitochondrial oxphos gene expression levels in the presence and absence of rapamycin, as determined by qRT-PCR. Data represent the mean ± SD of three independent experiments. *P < 0.05, **P < 0.01, and ***P < 0.001

We next evaluated the effects of rapamycin on mitochondria in NIH3T3 cells. The cells were treated with or without rapamycin and immunostained with MitoTracker. Rapamycin decreased the MFI (rapamycin 1 nM 69 ± 6 MFI vs. Nil 124 ± 24.26 MFI; *P < 0.05) (Fig. 2a) and the mitochondrial membrane potential in NIH3T3 cells MFI (rapamycin 1 nM 5446.4 ± 3176.1 MFI vs. Nil 2878 ± 1277.2 MFI; **P < 0.01) (Fig. 2b). As mitochondria produce ATP [17], we assessed the effects of rapamycin on oxygen consumption. The basal, ATP-linked, and maximal respiratory rates and reserve capacity were all lower in the rapamycin-treated group than in the control group (basal respiration: rapamycin 1 nM 64.64 ± 0.78 pmol/min vs. control 71.03 ± 0.35 pmol/min, ATP: rapamycin 1 nM 36.15 ± 0.78 pmol/min vs. control 42.68 ± 0.35 pmol/min, maximal respiration: rapamycin 1 nM 130.67 ± 2.96 pmol/min vs. control 166.68 ± 4.18 pmol/min, reserve capacity: rapamycin 1 nM 66.03 ± 2.96 pmol/min vs. control, 95.65 ± 4.18 pmol/min; *P < 0.05, **P < 0.01) (Fig. 2c). Rapamycin also decreased the expression levels of the mitochondrial oxidative phosphorylation–related genes *NDUFB5*, *UQCRB*, and *COX5B* (*NDUFB5*: rapamycin 10 nM 0.66 ± 0.03 vs. Nil 1.00 ± 0.15, *UQCRB*: rapamycin 10 nM 0.46 ± 0.04 vs. Nil 1.00 ± 0.12, *COX5B*: rapamycin 10 nM 0.46 ± 0.04 vs. Nil 1.00 ± 0.12; *P < 0.05) (Fig. 2d). These data indicate that rapamycin induces mitochondrial dysfunction.

### Rapamycin–metformin suppresses mitochondrial dysfunction

**Fig. 3:**
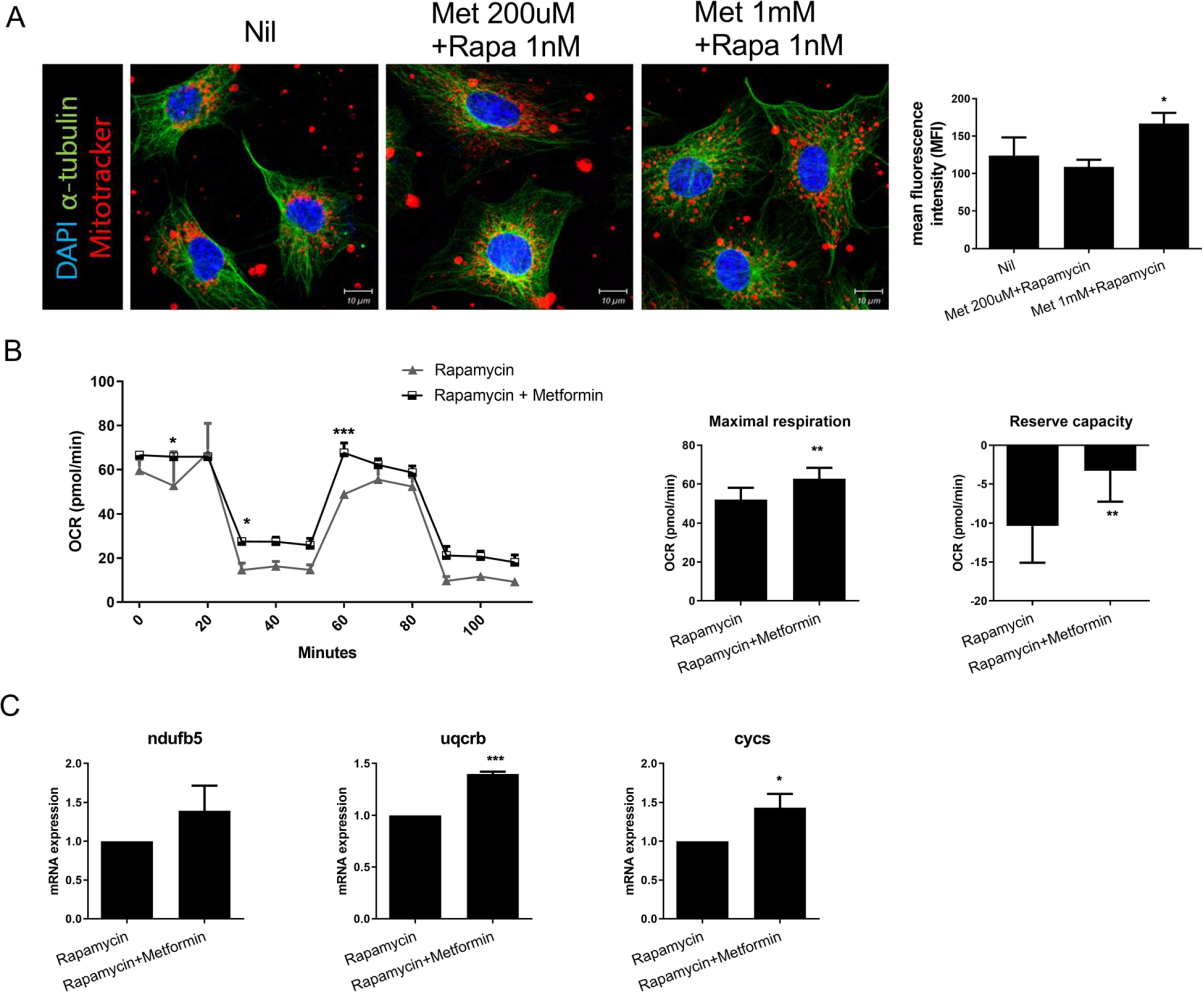
Metformin rescues rapamycin-induced mitochondrial dysfunction. **a** NIH3T3 cells were immunostained with MitoTracker Red CMXROS (red), anti-α-tubulin (green), and DAPI (nuclei, blue), and the perinuclear mitochondrial distance was quantified on confocal micrographs. **b** Cellular OCRs in the presence of rapamycin–metformin. Oligomycin 2 μM, FCCP 0.3 μM, metformin 2 mM, antimycin A 5 μM, and rotenone 5 μM were treatment in cells for OCR measurement. **c** Mitochondrial oxphos gene expression levels in the presence of rapamycin–metformin, as determined by qRT-PCR. Data represent the mean ± SD of three independent experiments. *P < 0.05, **P < 0.01, and ***P < 0.001

Metformin directly inhibits mitochondrial complex I and increases the uncoupled respiration ratio [18]. We investigated the effects of rapamycin–metformin on mitochondrial function. MitoTracker staining showed that the MFI of rapamycin–metformin-treated NIH3T3 cells was greater than that of control cells (metformin + rapamycin 166.94 ± 14.13 vs. Nil 124 ± 24.26; *P < 0.05) (Fig. 3a). The maximal respiratory rate and reserve capacity of NIH3T3 cells were also increased by rapamycin–metformin (maximal respiration: rapamycin + metformin 62.78 ± 5.59 pmol/min vs. rapamycin 52.19 ± 5.94 pmol/min, reserve capacity: rapamycin + metformin 1 nM − 3.29 ± 3.94 pmol/min vs. rapamycin − 10.37 ± 4.72 pmol/min; **P < 0.01) (Fig. 3b). In addition, the mRNA levels of *NDUFB5*, *UQCRB*, and *CYCS* were increased by rapamycin–metformin compared with rapamycin monotherapy (*UQCRB*: rapamycin + metformin 1.4 ± 0.02 vs. rapamycin 1.00 ± 0, *COX5B*: rapamycin 10 nM 1.43 ± 0.18 vs. rapamycin 1.00 ± 0; *P < 0.05, ***P < 0.001) (Fig. 3c). Our results suggest that rapamycin–metformin reversed rapamycin-induced mitochondrial dysfunction.

### Anti-inflammatory effects of rapamycin–metformin

**Fig. 4:**
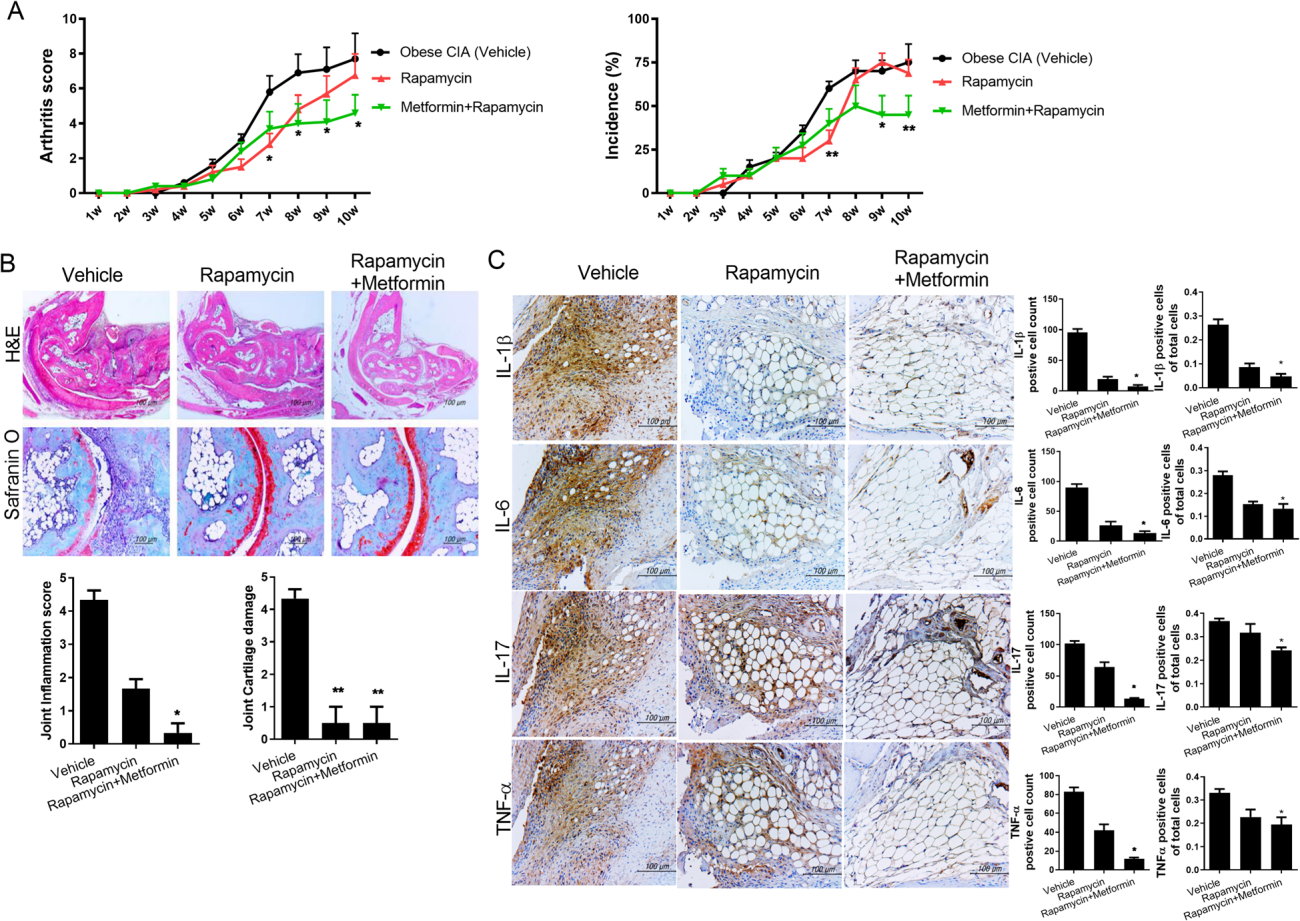
Preventive effect of rapamycin–metformin combination in obese mice with collagen-induced arthritis (CIA). **a** Rapamycin–metformin reduced the arthritis score and incidence. **b** Histological features of joints; images were stained with hematoxylin and eosin and safranin O. Original magnifications, × 40 and × 200. **c** IL-1β, IL-6, IL-17, and TNF-α levels in joint-tissue sections. Each cytokine-positive cells in the total cell number were counted and graphed. Original magnification, × 400. In vivo animal experiments were conducted five times per group. Tissues were analyzed from five mice of each group. Data represent the mean ± SD. *P < 0.05 and **P < 0.01

To further investigate the therapeutic effects of drug combination on RA, mice with CIA that were fed the HFD for 10 weeks were used [19]. Mice were administered rapamycin (1 mg/kg/day), metformin (50 mg/kg/day), rapamycin–metformin (rapamycin 1 mg/kg/day + metformin 50 mg/kg/day), or vehicle (saline) on day 7 after the first immunization. Rapamycin–metformin treatment attenuated the arthritis score and decreased the incidence rate compared with the controls (8W arthritis score: rapamycin + metformin 4 ± 3.59 vs. obese CIA 6.9 ± 3.38, 9W arthritis score: rapamycin + metformin 4.1 ± 3.96 vs. obese CIA 7.1 ± 3.98, 10W arthritis score: rapamycin + metformin 4.6 ± 3.31 vs. obese CIA 7.7 ± 4.64, 9W incidence score: rapamycin + metformin 45 ± 34.96 vs. obese CIA 70 ± 19.72, 10W incidence score: rapamycin + metformin 45 ± 34.96 vs. obese CIA 75 ± 33.33; *P < 0.05, **P < 0.01) (Fig. 4a). Histological analyses revealed that joint inflammation and cartilage damage were reduced in mice treated with rapamycin–metformin compared with the controls (joint inflammation score: rapamycin + metformin 0.33 ± 0.29 vs. vehicle 4.33 ± 0.29, joint cartilage damage: rapamycin + metformin 0.5 ± 0.5 vs. vehicle 4.33 ± 0.29; *P < 0.05, **P < 0.01) (Fig. 4b). The levels of IL-1β, IL-6, IL-17, and TNF-α, which are associated with chronic joint inflammation and tissue destruction [20], were significantly suppressed in the joints of rapamycin–metformin-treated mice (IL-1β: rapamycin + metformin 7.33 ± 2.52 vs. vehicle 95.67 ± 5.51, IL-6: rapamycin + metformin 13.67 ± 3.21 vs. vehicle 90.33 ± 5.51, IL-17: rapamycin + metformin 13.33 ± 1.53 vs. vehicle 102 ± 4, TNF-α: rapamycin + metformin 11.67 ± 1.53 vs. vehicle 83 ± 4.58; *P < 0.05) (Fig. 4c).

### Reciprocal regulation of Th17/Treg cells by rapamycin–metformin

**Fig. 5:**
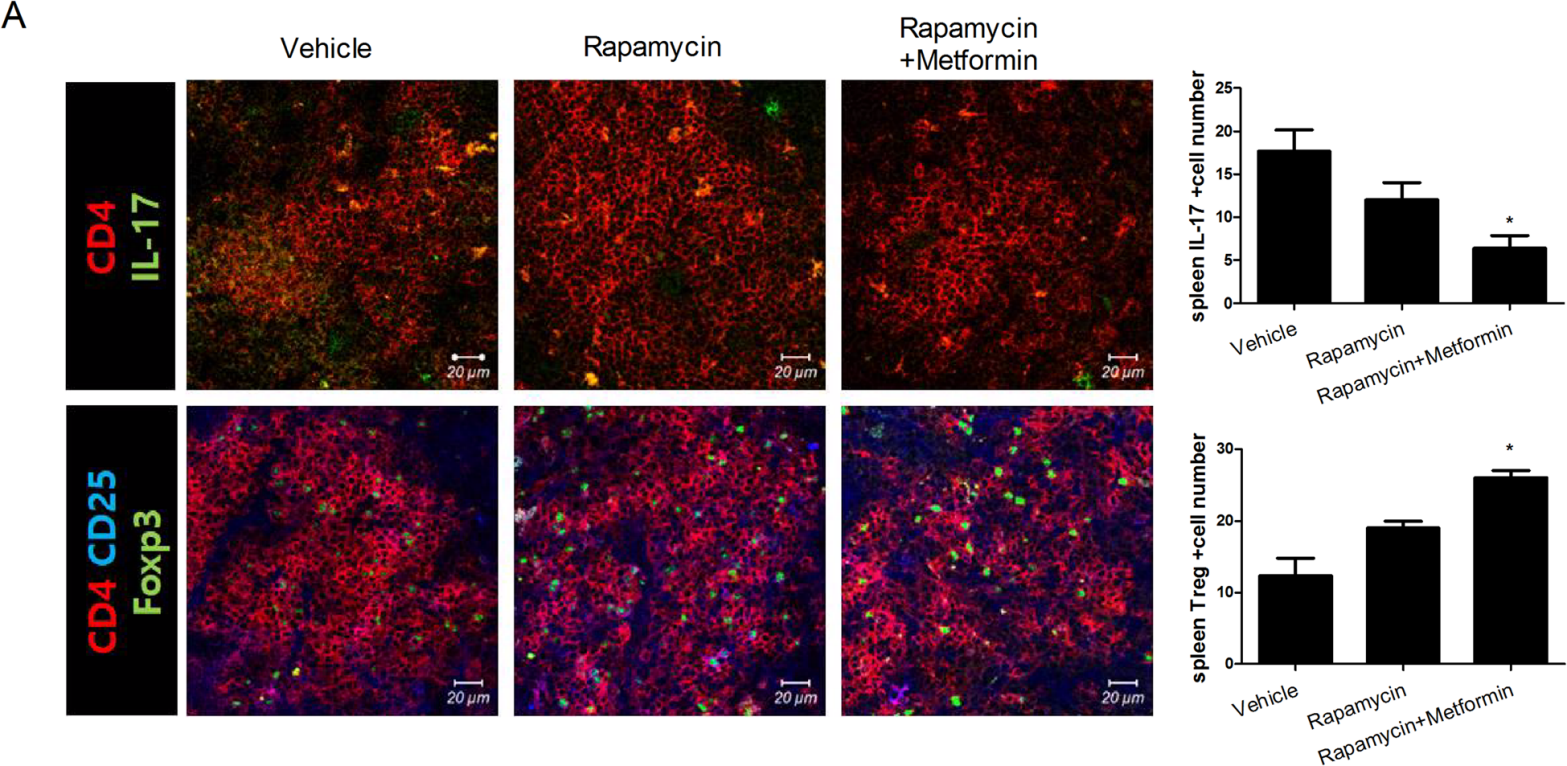
Regulation of Th17/Treg cells in obese mice with CIA by rapamycin–metformin treatment. **a** Rapamycin–metformin decreased the frequency of Th17 cells within the CD4^+^ T cell population and induced Treg production in the spleen tissues of obese mice with CIA. Original magnification, × 400. Tissues were analyzed from five mice of each group. Data represent the mean ± SD. *P < 0.05

Next, we investigated the effects of rapamycin–metformin on the splenic populations of Th17 and Treg cells by confocal microscopy. The number of CD4^+^IL-17^+^ cells was reduced and that of CD4^+^CD25^+^Foxp3^+^ cells was increased in rapamycin–metformin-treated mice compared with the controls (CD4^+^IL-17^+^ cells: rapamycin + metformin 6.33 ± 1.53 vs. vehicle 17.67 ± 2.52, CD4^+^CD25^+^Foxp3^+^ cells: rapamycin + metformin 26 ± 1 vs. vehicle 12.33 ± 2.52; *P < 0.05) (Fig. 5a). Thus, rapamycin–metformin attenuated Th17/Treg imbalance in mice with CIA.

### Effects of rapamycin–metformin on the metabolic profile

**Fig. 6:**
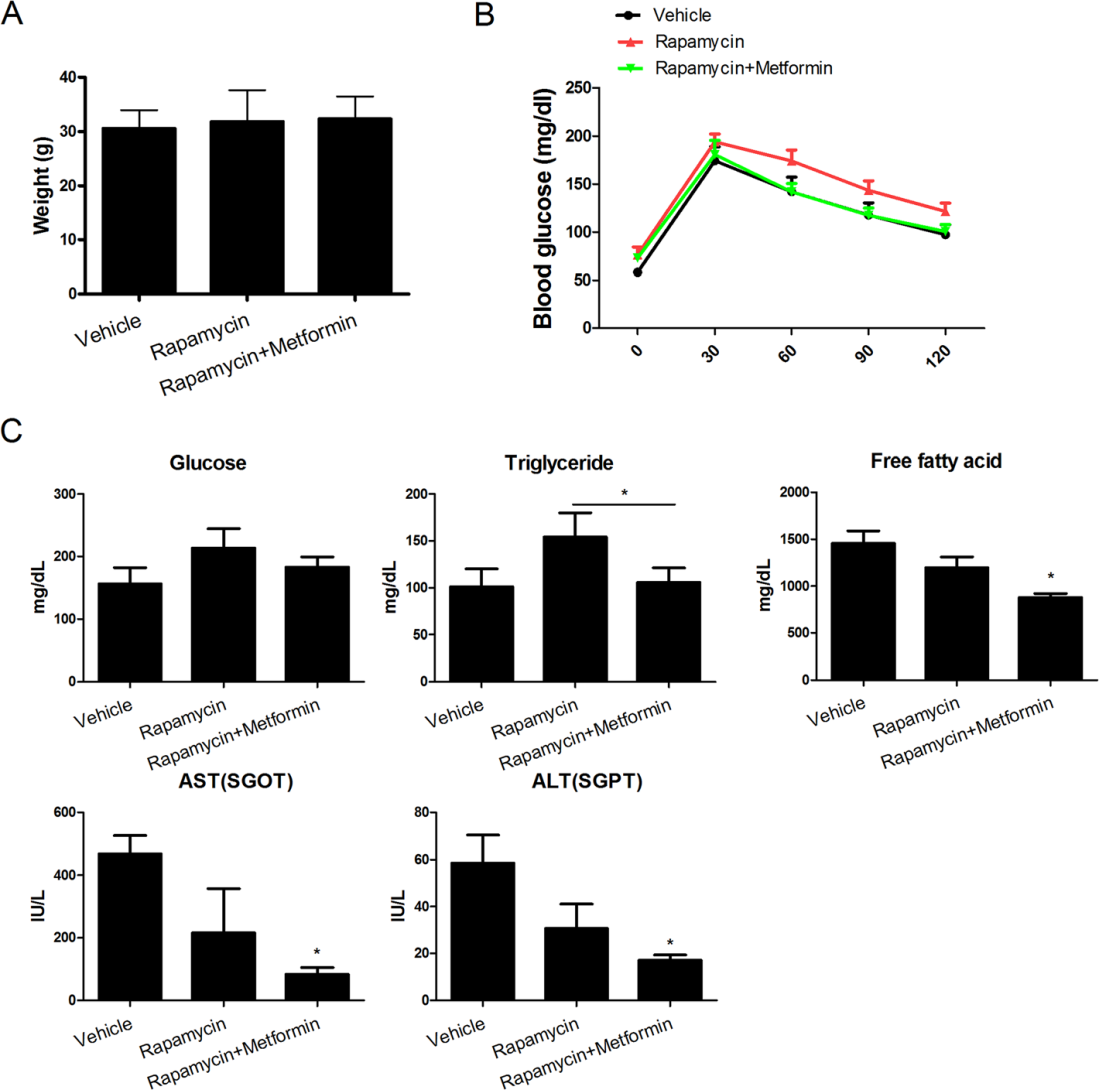
Combination of rapamycin–metformin can suppress the metabolic profile of obese mice with CIA. **a** Liver weights. **b** Results of glucose and insulin tolerance tests. **c** Metabolic profiles. Data represent the mean ± SD of three independent experiments. *P < 0.05 and ***P < 0.001

As RA is related closely to metabolic dysfunction, we assessed the effects of rapamycin–metformin on the metabolic profile of mice with CIA. The body weight was unaffected by rapamycin–metformin (Fig. 6a). Rapamycin–metformin-treated obese mice with CIA had lower blood insulin resistance levels than did those treated with rapamycin alone (Fig. 6b). Rapamycin increased the serum levels of glucose (Fig. 6c) and triglycerides, but the addition of metformin decreased these levels. In addition, the serum levels of free fatty acids, AST, and ALT were lower in rapamycin–metformin-treated obese mice with CIA than in the controls (free fatty acids: rapamycin + metformin 877.4 ± 41.71 vs. vehicle 1453.5 ± 95.5, AST: rapamycin + metformin 83.4 ± 21.73 vs. vehicle 468.5 ± 41.5, ALT: rapamycin + metformin 17 ± 2.35 vs. vehicle 58.5 ± 8.5; *P < 0.05) (Fig. 6c). Therefore, rapamycin–metformin improved the metabolic profile of obese mice with CIA.

## Discussion

The mTOR inhibitor rapamycin is an immunosuppressive agent used to prevent acute rejection in renal transplant recipients by activating autophagy via the mTOR pathway [21], and several mTOR inhibitors are used in the treatment of some solid tumors [22]. In addition, some studies have demonstrated the therapeutic potential of mTOR inhibitors in RA [11, 12]. However, rapamycin has the intrinsic limitations that it can lead to metabolic disorders and mitochondrial dysfunction [7, 8]. This study was performed to investigate whether a combination of rapamycin and metformin could regulate the inflammatory response in RA while minimizing mitochondrial dysfunction as a side effect of rapamycin. In the present study, rapamycin monotherapy reduced T cell alloreactivity and the levels of proinflammatory cytokines and induced the expression of Foxp3^+^Treg cells, in vitro. In vivo, rapamycin monotherapy also exerted an anti-inflammatory effect by reducing the expression of proinflammatory cytokines and suppressed the Th17 population while increasing the Treg population. However, rapamycin monotherapy intensified mitochondrial dysfunction, with features such as decreased membrane potential and respiration, as described previously [7, 8]. Mitochondria are important for the control of inflammation because they regulate cell death, act as master regulators of intracellular and extracellular danger signaling, and are involved in various metabolic pathways [23, 24]. Mitochondrial dysfunction can be caused by oxidative stress and viral infection, or by mitochondrial damage, and has been suggested to play a role in the pathogenesis of various diseases, including RA, neurodegenerative diseases, and metabolic disorders [10, 23, 25, 26]. Mitochondrial dysfunction was found to decrease the apoptosis of FLSs in patients with RA and to aggravate the inflammatory response of human synoviocytes [10, 25]. We attenuated rapamycin-induced mitochondrial dysfunction with metformin, and the present study yielded the promising result that the combination of rapamycin and metformin attenuated inflammation in RA while maintaining mitochondrial function.

Metformin is an effective antidiabetic and antiobesity agent that also exerts anti-inflammatory effects via the AMPK-mTOR pathway, a downstream signaling pathway of the mitochondrial respiratory chain complex, and modulates intracellular oxygen redistribution [15]. Indeed, combined treatment with rapamycin and metformin inhibited tumor growth in obese prediabetic mice [27]. We reported previously that metformin improves obesity and metabolic dysfunction by activating fibroblast growth factor 21 and regulating Th17/Treg imbalance [28]. Therefore, we hypothesized that the addition of metformin to rapamycin would improve mitochondrial dysfunction and the metabolic profile while suppressing CIA. In the present study, the combination of rapamycin–metformin showed beneficial effects on CIA and improved mitochondrial respiration and membrane potential. Surprisingly, rapamycin–metformin upregulated the expression of the ATP-related gene *NDUFB5* and the complex I genes *UQCRB* and *COX5B* in NIH3T3 cells. Moreover, rapamycin–metformin regulated inflammation and mitochondrial function in the mouse spleen (data not shown). Furthermore, rapamycin–metformin mitigated arthritis and cartilage degradation in obese mice with CIA by reducing proinflammatory cytokine expression in affected joints and regulating Th17/Treg imbalance. Increased Th17 populations and decreased Treg populations are known to play important pathological roles in autoimmune diseases, including RA [29, 30]. Here, we report that the rapamycin–metformin combination effectively attenuated autoimmunity via regulation of Th17/Treg imbalance and suppression of proinflammatory cytokines.

Metabolic syndrome is more common in patients with RA than in healthy controls [31], and management of the combined risk factors of cardiovascular diseases, such as metabolic syndrome, is important due to the increased risk of cardiovascular disease in patients with RA [32]. Furthermore, obesity is known to decrease the response rate to TNF-α inhibitors in patients with RA [33]. Therefore, the management of metabolic syndrome and obesity in patients with RA is important with regard to cardiovascular disease and drug responsiveness. In the present study, levels of free fatty acids and aminotransferases were elevated by the HFD and were reduced effectively by the combination of rapamycin and metformin. Therefore, rapamycin–metformin can not only dampen the inflammatory response, but also improve the metabolic profile in obese mice with CIA.

## Conclusions

In conclusion, we reported here that rapamycin–metformin ameliorates arthritis and inflammation in vitro and in vivo while not inducing mitochondrial dysfunction, a potential side effect of mTOR inhibitors. Furthermore, the metabolic disorder-related elevation of free fatty acid and aminotransferase levels was improved by this drug combination. These beneficial effects suggest that the rapamycin–metformin combination is a potential therapeutic option for RA patients with obesity, as it increases the therapeutic potential of rapamycin while minimizing its side effects.

## Data Availability

Not applicable

## Abbreviations

RA: Rheumatoid arthritis
CIA: Collagen-induced arthritis
FLSs: Fibroblast-like synoviocytes
AST: Aspartate aminotransferase
ALT: Alanine aminotransferase
AMPK: AMP-activated protein kinase
mTOR: Mammalian target of rapamycin
Th17: T helper 17 cell
Treg: Regulatory T cell
IL-17: Interleukin-17
IL-6: Interleukin-6
TNF-α: Tumor necrosis factor-α
NDUFB5: NADH dehydrogenase 1 beta subcomplex, 5
UQCRB: Ubiquinol-cytochrome C reductase binding protein
COX5B: Cytochrome C oxidase subunit 5B
